# Preoperative CT Voiding Cystourethrography Using 16-Multidetector CT in Female Urethral Diverticulum

**DOI:** 10.1371/journal.pone.0107448

**Published:** 2014-09-12

**Authors:** Young Ju Lee, Seung Jun Son, Jae-Seung Paick, Soo Woong Kim

**Affiliations:** Department of Urology, Seoul National University Hospital, Seoul, Korea; Affiliated Hospital of North Sichuan Medical College, China

## Abstract

**Purpose:**

To evaluate the clinical usefulness of preoperative CT voiding cystourethrography (CT-VCUG) using 16-multidetector computed tomography for female urethral diverticula.

**Materials and Methods:**

Preoperative CT-VCUG was performed in 15 consecutive patients who underwent urethral diverticulectomy from May 2004 to December 2012. The result of preoperative cystourethroscopy and surgical findings were recorded by a single surgeon and CT-VCUG findings including the location of osita were retrospectively reviewed by another urologist who was blinded to the surgical finding. The location of the ostium detected on CT-VCUG was compared descriptively with the intraoperative surgical and preoperative cystourethroscopic findings.

**Results:**

A total of 14 consecutive patients who underwent preoperative CT-VCUG and urethral diverticulectomy were included in the analysis. Ostia were detected on CT-VCUG in all cases, whereas ostia were identified in 10 patients (71.4%) by cystourethroscopy. Ostia were located between the 4 and 8 o’clock direction. Mean distance from the bladder neck to the ostium was 24.2 mm. Circumferential and horseshoe shaped diverticula were observed in 6 and 4 patients, respectively. The surgical findings correlated well with the CT findings.

**Conclusions:**

Preoperative CT-VCUG can be useful in identifying the ostia of urethral diverticula in patients scheduled for urethral diverticulectomy and can provide structural information, useful to establish surgical strategy.

## Introduction

Female urethral diverticulum is a localized outpouching of the urethra into the anterior vaginal wall. Acquired diverticulum may arise from repeated infections with abscess formation within the periurethral and urethral glands [1], affecting 0.6–6% of women, mostly in their third to fifth decade [2]–[4]. It is one of a challenging topic in the field of diagnostic and reconstructive urology.

The diagnosis of urethral diverticulum is often delayed and difficult because most of the patients present with nonspecific genitourinary symptoms such as dysuria and recurrent urinary tract infections [5]–[7]. A few patients present with the classic triad of symptoms, which consisted of dysuria, postvoid dribbling, and dyspareunia [4], [8]. Therefore, a high index of suspicion is important for the diagnosis of urethral diverticulum.

With the advance of technology, imaging plays more determinant role for the diagnosis of urethral diverticulum than symptoms alone [4]. Voiding cystourethrography (VCUG), urethrography, ultrasonography, computed tomography (CT), magnetic resonance imaging (MRI), and cystourethroscopy have been used as a diagnostic modality [8], [9]. No one modality has been established as the gold standard of diagnosis, and none of them has perfect sensitivity to visualize the ostium, which is indispensable in surgical planning [9]. Few studies have been conducted to evaluate the usefulness of CT in female urethral diverticulum [10], [11]. CT-VCUG has an advantage in clear visualization of the ostium [10].

Herein, we evaluated the usefulness of CT-VCUG as a preoperative imaging in females with urethral diverticula.

## Materials and Methods

After obtaining an approval of institutional review board, retrospective review was performed for 16 consecutive patients who underwent urethral diverticulectomy by a single surgeon in our institution between March, 2004 and December, 2012. Fifteen patients underwent preoperative CT-VCUG. As one patient could not complete CT-VCUG because of the voiding issue, 14 patients who completed CT-VCUG were included in the analysis. Age at operation, presenting symptoms, the causes of operation which the surgeon had mentioned in medical records, initial diagnostic modality, the result of CT-VCUG, preoperative cystourethroscopic and intraoperative surgical findings regarding the location of the diverticular ostia were retrospectively reviewed. Operative findings regarding the ostia were determined by the surgeon and were recorded in the operative records.

Data regarding the CT-VCUG finding was retrospectively reviewed by a urologist who was blinded to the surgical findings. The shape of the diverticulum and the presence of diverticular ostia were evaluated. If there was an ostium, the direction of it and the distance from the bladder neck to the ostium were recorded. The presence of diverticular ostia was determined if the communication between the diverticulum and the urethra was visualized by the presence of contrast media in either axial or coronal image. The direction of the diverticular ostium was defined as the ventral side 12 o’clock position on the axial image. The distance between the bladder neck and the urethral opening was estimated with multiplication of the thickness of scan and the number of stacks. The shape of the diverticulum was categorized as simple, horseshoe and circumferential.

The protocol of CT-VCUG was same as we previously reported in our preliminary study [11]. To describe it briefly, the bladder was filled with 300 mL of contrast medium, and then scanning with a 16-multidetector CT was performed in the supine position from the top of the bladder to the inferior margin of the symphysis pubis. After the initial pre-void scan, patients were instructed to give hand signals on voiding to take void and post-void scans. Less than 7 seconds were required for each scan, which made it possible to capture the image during voiding.

Preoperative cystourethroscopy was performed under general anesthesia at the time of surgery by the same surgeon. The surgical procedure of urethral diverticulectomy was as follows. After an inverted U-shaped incision of the anterior vaginal wall, periurethral fascia was opened longitudinally. Diverticular sac was mobilized and dissected to reveal its communication with the urethra and excised completely. The urethral defect was closed longitudinally over a Foley catheter with continuous or interrupted absorbable sutures. Martius labial fat pad graft was placed if indicated. If there was no symptomatic recurrence, further follow-up visits were not made after 3 months postoperatively.

The results of preoperative CT-VCUG were compared with operative and cystourethroscopic findings, in regard of diverticular ostia. Due to the small number of subjects, statistical analysis was not necessary.

### Ethics Statement

This research was approved by the institutional review board of Seoul National University Hospital and conducted following the principles as expressed in the Declaration of Helsinki. Written informed consent was exempted and approved by the institutional review board because this was a retrospective study. Patient records were anonymized and de-identified prior to the analysis.

## Results

The mean age was 41 (range 28–57) years. Dysuria was the most common symptom and present in all patients. Eight patients had a history of recurrent cystitis. Only 3 patients (21.4%) had classic triad of symptoms. Three patients had a previous history of urethral diverticulectomy and 1 patient had undergone an incision and drainage before referral to our center. Because this was a tertiary hospital, all patients were tertiary referrals from other hospitals with a suspicious or confirmed diagnosis. Diverse modalities were used to make an initial diagnosis. VCUG (n = 5), CT (n = 4), transvaginal ultrasound (n = 2), cystourethroscopy (n = 2), intravenous pyelography (n = 1) and MRI (n = 1) were used.

**Figure 1:**
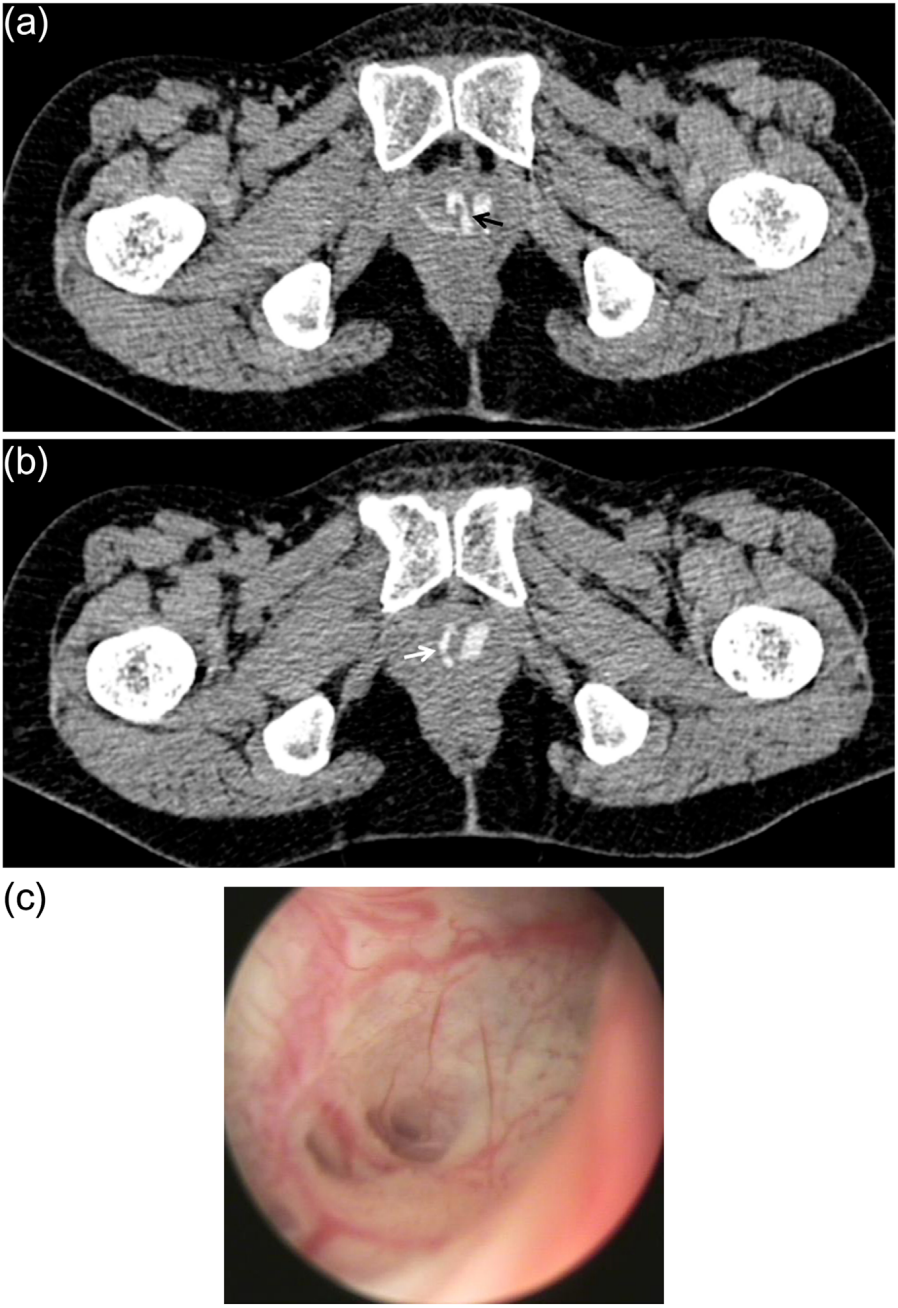
Comparison of CT-VCUG and cystourethroscopy showing two ostia. Forty-nine year old female presented with recurrent cystitis. The axial image of CT-VCUG showed the diverticular ostia (a) at 5 (black arrow) and (b) 7 o’clock (white arrow) direction. (c) Cystourethroscopy of the same patient revealed an opening under direct vision at the location as predicted by CT-VCUG.

Patients’ characteristics are described in Table 1. Six patients (42.9%) had circumferential shaped diverticula. Horseshoe shaped and simple diverticula existed in 4 patients, respectively. The ostia could be identified on preoperative CT-VCUG in all patients, whereas preoperative cystourethroscopy detected the ostia in 10 patients (71.4%). The direction of ostia lied between 4 and 8 o’clock position and the majority (75%) were between 5 and 7 o’clock position. Mean distance from the bladder neck to the ostium was 24.2 mm (range 14–33). Two patients had multiple ostia detected on CT-VCUG. Figure 1 shows the image of CT-VCUG and cystourethroscopy of a patient, presented with two ostia in 5 and 7 o’clock position. Operative findings were in accordance with the results of CT-VCUG.

**Table 1:** Characteristics of the patients.

Age atoperation	Chief complaintnecessitating imaging	Shape of urethraldiverticulum	Location of the ostium (O’ clock)		Follow-up	
			CT-VCUG	cystourethroscopy	Duration(month)	Symptomaticrecurrence
39	Frequency, dysuria	Circumferentialhorseshoe	5	5	95	−
40	Recurrent dysuria and pyuria	Circumferentialhorseshoe	5	Fail	66	+^1^
46	Urethral pain, Bloody discharge	Simple cystic	5	5	4	−
57	Microscopic hematuria, urinary incontinence	Circumferentialhorseshoe	5	5	54	+^2^
49	Recurrent cystitis	Circumferentialhorseshoe	5,7	5,7	4	−
49	Urinary incontinence,recurrent cystitis	Simple cystic	6	6	26	−
38	Tender vaginal mass	Partial horseshoe	5,7,8	5	4	−
47	Urethral discomfortwith large amount of discharge	Partial horseshoe	7	7	14	−
32	Recurrent cystitis after delivery	Simple cystic	4	Fail	19	−
29	Acute pyelonephritis	Partial horseshoe	8	Fail	11	−
44	Referred patient fordysuria after urethral diverticulectomy	Partial horseshoe	7	7	9	−
39	Recurrent cystitis	Circumferentialhorseshoe	5	5	4	−
42	Recurrent cystitis	Circumferentialhorseshoe	7	Fail	3	−
28	Vaginal bulging mass with dysuria	Simple cystic	7	7	3	−

1 Redo urethral diverticulectomy at postop 61 months.

2 Redo urethral diverticulectomy at postop 47 months.

Neither intradiverticular calculi nor urethral carcinoma was found in any of them. Three patients (21.4%) underwent urethral diverticulectomy with Martius graft, whereas all the others underwent simple urethral diverticulectomy. None of them had a symptomatic recurrence at 3 months after surgery. Two of them had recurrence during follow-up and underwent corrective surgery at 47 and 61 months after surgery.

## Discussion

Identification of an ostium and the complete closure of it is a crucial procedure in urethral diverticulectomy. Therefore, getting an information about the location of an ostium is useful to establish a preoperative strategy. The present study showed that preoperative CT-VCUG visualized the communication between the urethral diverticulum and the urethra in all cases. The operative finding regarding the ostium was in agreement with the result of CT-VUCG. The sensitivity of cystourethroscopy was 71.4%, which was similar to the previous reports of 15–77% [5], [12].

Although CT-VCUG and VCUG share the same concept of taking images during voiding, the two modalities are different. VCUG have a high false negative rate, which is attributed by incomplete distension of the diverticulum secondary to poor urinary flow rates, loculations, or narrow diverticular ostia [13]. CT-VCUG has several advantages over VCUG. By the virtue of advances in imaging technology, fast scan with high resolution is possible. This provides more detailed structural information including the communication between the urethra and the diverticulum, as we previously had shown in two patients [11]. The high resolution of CT made it feasible to detect the small amount of contrasts between the urethra and the diverticulum, even in conditions of incomplete distension of the large diverticula or narrow ostia due to concurrent edema or inflammation.

**Figure 2:**
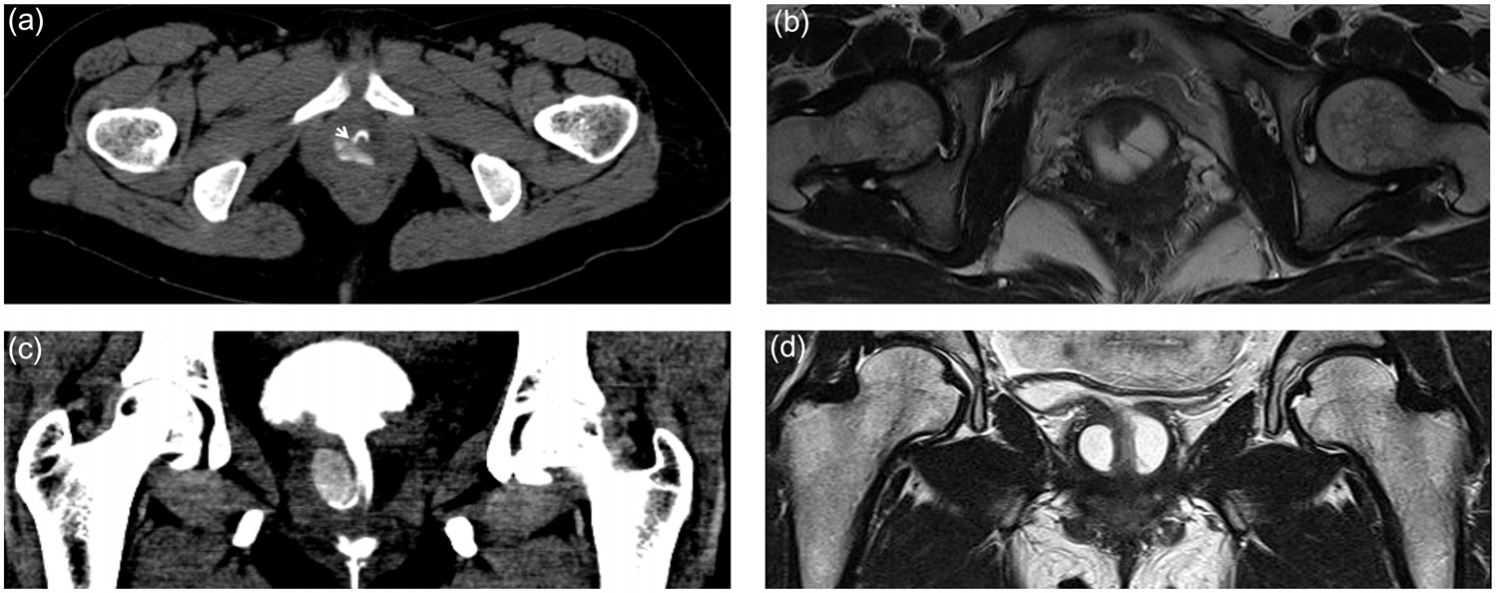
Comparison of CT-VCUG and MRI in a 42 year-old female. (a) The axial image of CT-VCUG of voiding phase showed a diverticulum partially filled with contrast media. (b) The axial image of T2 weighted MRI demonstrated the overall shape and complexity of the urethral diverticulum. (c) Coronal image of CT-VCUG revealed the ostium clearly. (d) Coronal image of T2 weighted MRI revealed suspicious ostium, but not as clearly as in CT-VCUG.

MRI provides excellent soft-tissue contrast, which allows the delineation of the urethra and its supporting structures, having a strength in visualizing the overall shape of a diverticulum. Recently, MRI has become the diagnostic imaging of choice for periurethral lesions. Some urologists regard MRI as the gold standard for diagnosis with a sensitivity of 100% [14], [15]. However, Chung et al. reported that in cases of clinically suspicious urethral diverticula, 24.4% of patients showed substantial discrepancy between the operative findings and the results of MRI. In their study, 2 patients had a major discrepancy regarding the site or anatomy of urethral diverticulum, which made intraoperative decision difficult [16]. Furthermore, in a view of ostia, MRI does not provide excellent sensitivity. Portnoy et al. compared MRI with double balloon urethrography and reported that the ostium was detected in five of nine patients (55.5%) on each modality [17]. Ockrim et al. insisted that the most useful preoperative imaging is MRI for anatomical configuration and VCUG for detrusor/urethral function [15]. In their study, diverticular neck was identified on MRI in 4 of 11 patients, whereas 9 of 18 patients (50%) had a detectable ostium by VCUG. Dwarkasing et al. showed that the ostium was detectable by MRI with rigid endoluminal coil with a sensitivity of 85% [18]. In their study, the ostium was identified by preoperative cystourethroscopy in 8 of 17 cases (47%), which was lower than that of our result. Figure 2 depicts the comparison of MRI and CT-VCUG in the same patient. The strong point of CT-VCUG is that the ostium could be visualized in the dynamic process of voiding. The weak point is that the entire diverticulum might not be filled with contrast media if the size of diverticulum is very large, which might compromise the visualization of an entire diverticulum. In contrast to CT-VCUG, MRI does not include a voiding phase. The diverticular sac could be shrunk, which makes it harder to identify the communication between the urethra and the diverticulum.

Preoperative CT-VCUG could be beneficial to the surgical planning of horseshoe/circumferential shaped diverticula considering the level of surgical difficulty. Several previous studies have shown that the failure or recurrence was more likely for those with horseshoe or circumferential shaped diverticulum or previous pelvic surgery [15], [19], [20], and the complete excision of the diverticular sac is important especially in circumferential diverticula [20]. The present study included horseshoe or circumferential shaped diverticulum in 10 of 14 patients (71.4%), and the ostia could be addressed in all patients. Due to the limited number of patients, the inference might not be strong enough to have a solid conclusion. Larger number of patients with longer follow-up will be needed.

MRI is more expensive than CT and not all centers have MRI. Therefore, CT-VCUG could be more handy to do in many centers. However, CT-VCUG has a few disadvantages. First of all, this imaging modality carries ionizing radiation, which is a major discern of this modality. However, this could be reduced by modifying the protocol. The scanning height could be shortened from the top of the bladder neck to the urethral opening, and the voiding phase was enough to recognize the urethral structure. Second, if the diverticulum is large or complex and filled with pre-existing fluids, the contrast media cannot fill the lumen of diverticula to delineate the overall shape. However, a scanty amount of contrast media was enough to reveal the ostium of the urethral diverticulum. According to our experience, the detection of an ostium was not affected by the size or shape of the diverticulum. Lastly, the patient needs to void on the CT table to complete the study.

The present study has a few limitations. This study is a retrospective review and couldn’t provide the sensitivity and specificity of CT-VCUG for the diagnosis of urethral diverticulum because this was performed in a purpose of preoperative evaluation. And due to the small number of patients, statistical analysis couldn’t be made to compare the result with other imaging modalities. Because of this disadvantage, this study is descriptive in principal. Although a number of limitations exist, the present study is the first attempt to evaluate the usefulness of CT-VCUG in a series of patients with urethral diverticula.

In conclusion, preoperative CT-VCUG could locate the ostium of urethral diverticula in all patients and the surgical finding was in accordance with the result of CT-VCUG. Moreover, CT-VCUG provides structural information such as size and shape of the urethral diverticulum. This imaging modality is useful in surgical planning once the patient is determined to proceed with the surgical treatment.
